# Protective potential of *Angelica sinensis* polysaccharide extract against ethylene glycol-induced calcium oxalate urolithiasis

**DOI:** 10.1080/0886022X.2018.1496935

**Published:** 2018-11-06

**Authors:** Shengbao Wang, Xiaoran Li, Junsheng Bao, Siyu Chen

**Affiliations:** The Emergency Center, Gansu Nephro-Urological Clinical Center, Lanzhou University Second Hospital, LanzhouChina

**Keywords:** Urolithiasis, ethylene glycol, *Angelica sinensis* polysaccharide, reactive oxygen species, kidney injury molecule-1

## Abstract

**Purpose:** To evaluate a *Angelica sinensis* polysaccharide aqueous extract as a preventive agent in experimentally induced urolithiasis using *in- vitro* and *vivo* models.

**Material and methods:***Angelica sinensis* polysaccharide was investigated *in vitro* to determine its antilithiatic effects on the formation and morphology of calcium oxalate (CaOx) crystals and was analyzed *in vivo* to determine its ability to prevent CaOx urolithiasis in rats subjected to ethylene glycol-induced urolithiasis. Potassium citrate administration was used in the positive control group. The urolithiasis-related biochemical parameters were evaluated in the rats urine, serum and kidney homogenates. Kidney sections were subjected to histopathological and immunohistochemical analyses, and urolithiasis-related phospho-c-Jun NH2-terminal protein kinase and kidney injury molecule-1proteins were evaluated by Western blot analyses.

**Results:***Angelica sinensis* polysaccharide exhibited concentration-dependent inhibition of CaOx crystal formation. The *in vitro* assay revealed significant inhibition of crystal formation (6.99 ± 1.07) in the group treated with 4.0 mg/mL *Angelica sinensis* polysaccharide extract compared with the control group (58.38 ± 5.63; *p* < .05). *In vivo*, after treatment with ethylene glycol for 28 days, urinary oxidative stress, oxalate, creatinine, urea and urolithiasis-related protein were significantly increased (*p* < .05), except for serum oxidative stress (*p* > .05). The rats administered the extract of *Angelica sinensis* polysaccharide showed significantly decreased pathological change and CaOx deposition (*p* < .05) compared with the urolithiatic rats. Significantly reduced levels of urinary oxidative stress, oxalate, creatinine, urea and urolithiasis-related protein were observed in the *Angelica sinensis* polysaccharide treatment groups (*p* < .05) compared with the nephrolithic rats.

**Conclusion:** The results presented here suggest that *Angelica sinensis* polysaccharide has the potential to inhibit CaOx crystallization *in vitro* and may present anti-urolithiatic effects *in vivo*.

## Introduction

Urinary system stone disease is a frequent disease that can adversely affect quality of life. Various factors such as genetic factors, characteristics of the residential area and their effects on alcohol use, smoking, air pollution, dietary habits, metabolism and stress are considered to trigger its formation. The majority (approximately 80%) of all stones are mainly composed of calcium oxalate (CaOx) [1]. Over the past two decades, clinicians have made advances in the management of urolithiasis following several techniques including endo-urological stone removal and extracorporeal shock wave lithotripsy [2]. However, these invasive procedures do not prevent the recurrence of stones and are costly. Shock waves may cause complications such as inflammation, ischemia, renal fibrosis, subcapsular hematomas and decreased renal function [3].

Recently, a nomogram for the prediction of kidney stone recurrence was reported using data from the Rochester epidemiology project: the 10- and 15-year rates of symptomatic recurrence were 31 and 39%, respectively [4]. Medical therapy for preventing recurrence, such as thiazide diuretics and citrate preparations, is not consistently effective and may have adverse side effects that compromise its long-term use. Hence, there is a need to establish a medical treatment for preventing recurrent stone formation.

Several pharmacological and clinical studies of traditional medicinal plants used to treat urolithiasis have publicized their therapeutic potential in various *in vitro* and *in vivo* models. Furthermore, plants provide an inexpensive source of medicine for the majority of the world's population. Such medicines present minimal or no side effects and are considered safe; in addition, studies have indicated that various herbal plants such as *Flos carthami* [5], *Ipomoea eriocarpa* [6], *Costus spiralis* [7], *Cissampelos pareira* [8] and *Herniaria hirsute* [9], have been successfully proven as prophylactic and curative medicines for urolithiasis. All these reports suggest that herbal medicines may be a useful strategy for preventing renal stones.

Traditional Chinese medicines are rich sources of bioactive substances that can be used to pretreat or cure various types of human diseases [10]. Polysaccharides are polymeric carbohydrate macromolecules composed of long chains of monosaccharide units that are connected by various glyosidic linkages. Currently, an increasing number of studies have focused on polysaccharides extracted from different Chinese medicines due to their potential pharmacological activities. *Angelica sinensis* polysaccharide, a bio macro molecule isolated from the roots of *Angelica sinensis*, has drawn increasing attention in recent years for its various bioactivities, such as hematopoietic, immunomodulatory, hepatoprotective and antioxidant activities, among other effects [11–13].

Hence, the present study was initiated to evaluate whether *Angelica sinensis* polysaccharide has any preventive effect against calcium oxalate stones (compared with the effect of potassium citrate), using suitable *in vitro* crystallization methods and animal models.

## Materials and methods

### Plant materials

The dry roots of *Angelica sinensis* polysaccharide were collected from Dingxi (Gansu Province, China). Plant identification was graciously provided by members of the School of Pharmacy at Lanzhou University, according to the identification standard of the Pharmacopoeia of the People’s Republic of China. The extraction and purification of the polysaccharide were performed as previously described [14]. The sugar content of *Angelica sinensis* polysaccharide (MW 72.9 kD) was approximately 96.7% and the constituent monosaccharides were arabinose, glucose and galactose, with a molar ratio of 1:2.5:7.5 [15].

### Calcium oxalate (CaOx) crystallization assay

The assay was performed as previously described [16]. Stock solutions of 10.0 mM calcium chloride and 1.0 mM sodium oxalate that contained 200 mM sodium chloride and 10 mM sodium acetate were adjusted to pH 5.7. For the experiments, solutions containing the *Angelica sinensis* polysaccharide extract were mixed simultaneously with the solution containing the crystallization reagents before crystallization (0 h) to obtain a final *Angelica sinensis* polysaccharide concentration of 0.5, 1, 2 or 4 mg/mL. The same amount of distilled water was mixed to solutions containing the crystallization reagents at time 0 h and these solutions were treated as the blank control (0 mg/mL). Additionally, 1 mg/mL potassium citrate (positive control) was mixed to the solutions containing the crystallization reagents. All samples were maintained under 500-rpm agitation at 37 °C for 24 h.

### Test animals and housing

Fifty adult male Sprague-Dawley (SD) rats weighing between 200 and 220 g were used in this study. For four weeks, the rats were kept in 12 h of ideal lighting at the appropriate temperature and fed with standard pellet rat feed. All experimental protocols were approved by the Institutional Animal Ethics Committee and carried out in accordance with guidelines from the Committee for the Purpose of Control and Supervision of Experiments on Animal. The experiment was conducted in accordance with accepted standard guidelines for the care and use of animals in scientific research. After one week of acclimatization, the rats were equally divided into five study groups. Group 1 was used as the control group. Free access to drinking water containing 1% ethylene glycol was provided to the rats in the experimental groups (groups 2–5) to induce urolithiasis throughout the entire 28-day experimental period [17]. The rats in group 2 were treated as the urolithiatic group; the rats in group 3 were treated with potassium citrate at 2g/kg/day for 28 days as the positive control group; the rats in group 4: ethylene glycol-treated animals received the standard drug *Angelica sinensis* polysaccharide (80 mg/kg/day) for 28 days and the rats in group 5: ethylene glycol-treated animals received the standard drug *Angelica sinensis* polysaccharide (320 mg/kg/day) for 28 days. The *Angelica sinensis* polysaccharide was dissolved in distilled water and administered orally by metallic gavage needle.

### Collection and analysis of urine and serum

Urine samples (24 h) were collected on the 28th day by keeping the animals in an individual metabolic cage. The animals had free access to drinking water during the urine collection period; 0.04% sodium azide was added to the collected urine before the latter was stored at 4 °C to prevent bacterial growth. Parameters such as urinary calcium, uric acid, potassium and magnesium were estimated from the urine, using a Hitachi-7150 and Roche Omnic analyzer. Urinary oxalate and citrate were measured using commercially available kits (Biovision, Ltd., Milpitas, CA; Instruchemie, Ltd., Netherlands).

Serum was also obtained for a biochemical analysis at the end of the experiment. We drew blood samples from the tails of the rats, and blood creatinine and urea levels were measured. Malondialdehyde (MDA), superoxide dismutase (SOD) and catalase (CAT) in the blood and urine were measured using three kits (Jiancheng Bioengineering, Ltd., Nanjing, Jiangsu, China).Serum creatinine and blood urea nitrogen were estimated with kit-based methods, using an automatic biochemistry analyzer.

### Analysis of the renal homogenate

At the end of the experiment, the rats were sacrificed by cervical decapitation and the kidneys were excised and isolated. Furthermore, they were cleansed from extraneous tissue and rinsed in ice-cold physiological saline. A 0.1 g sample of renal tissue was mixed with nine volumes of cold phosphate buffer (pH 7.4) and then homogenized. The homogenates were centrifuged at 16 000 ×g for 5 min at 4 °C. The supernatants were analyzed for SOD, CAT and MDA using the above kits.

### Histopathology and renal crystal deposits

After sacrifice, a kidney from each group was rapidly dissected out, washed immediately with saline and fixed in 10% phosphate-buffered formalin. Paraffin-embedded specimens were cut into 5 μm-thick sections and stained with hematoxylin and eosin (H&E). The pathological alterations were assessed as follows: no visible lesions = 0; weak tubules dilation, tubulo-interstitial inflammatory infiltration and lesion area <20% = 1; tubules dilation, tubulo-interstitial inflammatory infiltration and lesion area <40% = 2 and severe tubules dilation, massive tubulo-interstitial inflammatory infiltration and lesion area >40% = 3 [18].

The renal crystal deposits in were graded as follows: no CaOx =0; CaOx deposits in the papillary tip =1; CaOx deposits in the corticomedullary junction =2 and CaOx deposits in the cortex =3. If the CaOx deposits at multiple sites, the points were combined to provide a total score for each pathological section.

### Immunohistochemical staining

Sections were deparaffinized and rehydrated using xylene and ethanol, respectively. Then, the sections were boiled at a controlled final temperature of 121 °C for 20 min in 10 mm citrate buffer solution (pH 6.0) for antigen retrieval. Endogenous peroxidase activity was blocked by immersion in a 0.3% hydrogen peroxide solution for 10 min. After rinsing in PBS (pH 7.2), 10% goat serum was applied for 1 h at room temperature to block any nonspecific reactions and incubated overnight at 4 °C with a polyclonal phospho-c-Jun NH2-terminal protein kinase (P-JNK) antibody (rabbit polyclonal to P-JNK, ab4821, Abcam, Cambridge, UK; 1:100) or a polyclonal kidney injury molecule (KIM)-1 antibody (rabbit polyclonal to KIM-1, Hopebiot; 1:500). The sections were washed with PBS three times, incubated with HRP-conjugated secondary antibody (Envision TM Detection Kit, GK500705, Gene Tech, San Francisco, CA) at 37 °C for 30 min and then washed three times with PBS. Finally, the sections were incubated with 3,3′-Diaminobenzidine in 0.05 mol/L Tris buffer (pH 7.6) containing 0.03% H_2_O_2_ for signal development and the sections were counterstained with 20% hematoxylin. The slides were dehydrated, cleared, cover slipped and evaluated. Each sample was incubated with an isotypic antibody dilution under the same experimental conditions as the negative control. The samples were then viewed under a light microscope.

### Western blot

The kidney tissue (100 mg) was homogenized in 1 mL of lysis buffer mixed with 10 µL of phenyl methane sulfonyl fluoride (100 mM) before centrifugation (12 000 ×g for 5 min) at 4 °C. The supernatants were collected to determine the protein concentration. The protein concentration was calculated with a bicinchoninic acid protein assay kit; 80 μg of proteins were separated by sodium dodecyl sulfate polyacrylamide gel electrophoresis and the proteins were electro transferred to Polyvinylidene difluoride (PVDF) membranes. Subsequently, the PVDF membrane was blocked in 5% fat-free milk for 2 h. Next, the PVDF membrane was incubated with the primary antibody against P-JNK or KIM-1 at 4 °C overnight. Subsequently, the PVDF membranes were washed and then incubated with horseradish peroxidase-labelled secondary antibody for 1 h. The membranes were washed three times for 5 min each with 1x Tris-buffered saline; after exposure and development, the protein expression was analyzed using a gel imaging analysis system. A glyceraldehyde 3-phosphate dehydrogenase (GAPDH) antibody served as a control for protein loading. The Western blot assay was performed in triplicate.

### Statistical analyses

The results are presented as the mean ± standard error of the mean. The differences among data were statistically analyzed using one-way analysis of variance (ANOVA) followed by the Tukey test to determine the level of significance using Prism, GraphPad version 5 (GraphPad Software Inc., La Jolla, CA). Differences between the data were considered significant at *p* < 0.05.

## Results

### In vitro *experiments*

**Figure 1.**
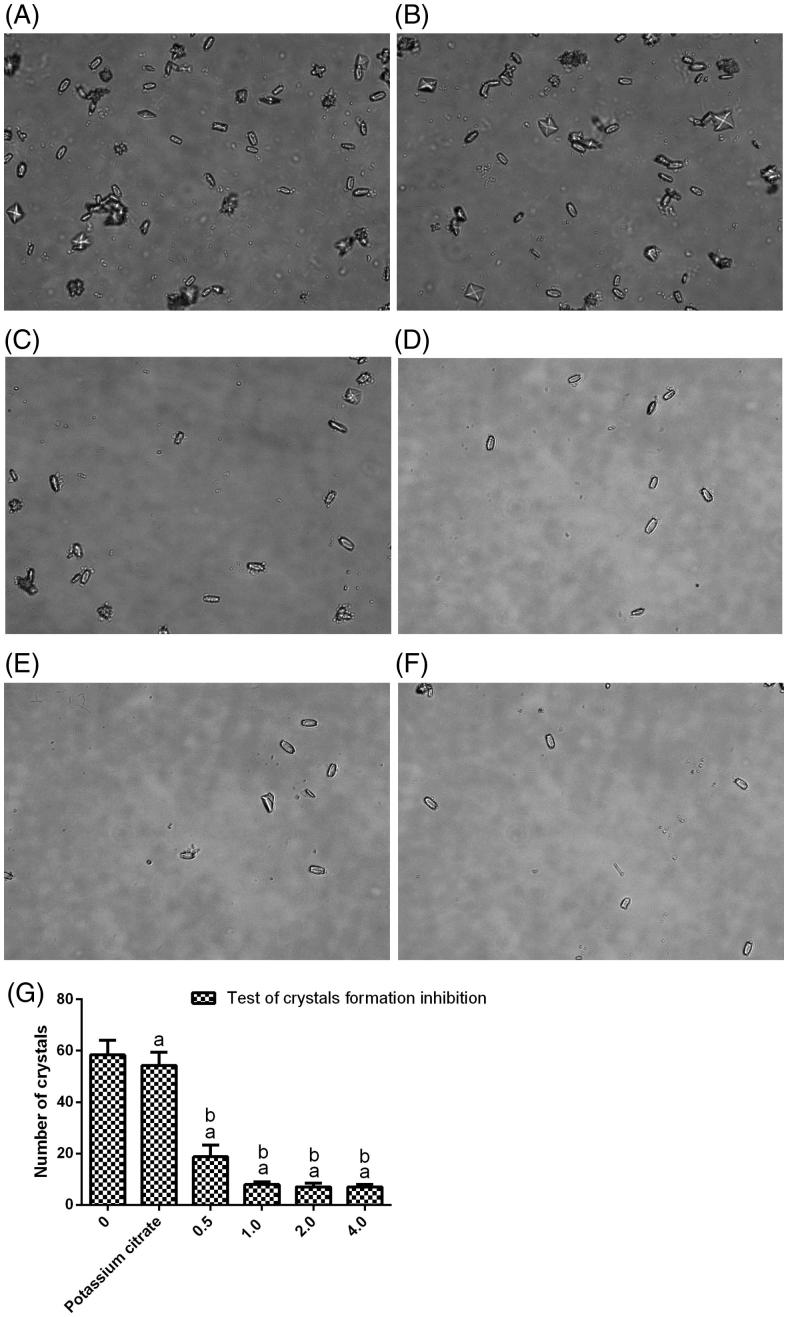
*In vitro* experiments to determine calcium oxalate crystallization after the administration of *Angelica sinensis* polysaccharide and potassium citrate (positive control).The CaOx crystals were observed under an inverted microscope (400×). (A) The blank control group exhibited crystal formation during the inhibition test. (B) 1mg/mL potassium citrate (positive control). (C–F)*Angelica sinensis* polysaccharide added to the solution containing the crystallization reagents at doses of 0.5, 1, 2 and 4 mg/mL, respectively. (G) Number of crystals in the crystal-formation inhibition tests. Values are expressed as the mean ± standard error. (a) *p* < .05 versus the blank control group (0 mg/mL *Angelica sinensis* polysaccharide) and (b) *p* < .05 versus the positive control group (1.0mg/mL potassium citrate).

The efficacy of *Angelica sinensis* polysaccharide in interfering with calcium oxalate crystal formation was observed in the crystallization assay. Crystallization was significantly inhibited with increases in the *Angelica sinensis* polysaccharide concentration from 0.5 to 1, 2or 4.0 mg/mL compared with that of the positive control or blank control (Figure 1(G)). These results showed the ability of *Angelica sinensis* polysaccharide to reduce the formation of calcium oxalate crystals.

### Urinary variables in different groups

Parameters recorded from the rat groups at the end of the 28-day treatment period are listed in Table 1. Significantly increased urine oxalate levels were observed in group 2 and were subsequently reduced by *Angelica sinensis* polysaccharide administration, which indicated that *Angelica sinensis* polysaccharide could reduce the oxalate level. In addition, compared with group 4, urine oxalate was significantly decreased in group 5 (*p* < .05).Urinary citrate was significantly increased in group 3 compared with group 2 (*p* < .05). However, the urinary citrate was not significantly improved in group 4 and group 5 compared with group 2. No significant difference was observed in the other urinary parameters compared to group 1.

**Table 1.** Changes in the urine values and serum parameters of the SD rats ($\bar{x}\pm s$).

Parameter	Group 1	Group 2	Group 3	Group 4	Group 5
Urine values					
Uric acid (mmol/L)	1.38 ± 0.21	1.40 ± 0.38	1.49 ± 0.13	1.51 ± 0.43	1.47 ± 0.37
Calcium (mmol/L)	2.91 ± 0.72	2.66 ± 0.24	2.71 ± 0.87	2.60 ± 1.05	2.79 ± 1.32
Oxalate (μmol/L)	102.3 ± 19.9	857.9 ± 96.5^a^	869.4 ± 88.3^a^	374.6 ± 37.0^abc^	190.1 ± 35.3^abcd^
Magnesium (μmol/L)	5.32 ± 1.33	5.11 ± 2.65	4.97 ± 1.31	5.67 ± 1.27	5.98 ± 0.74
Citrate (mmol/L)	0.86 ± 0.09	0.80 ± 0.21	6.89 ± 2.57^ab^	0.89 ± 0.09^c^	0.81 ± 0.25^c^
Potassium (mmol/L)	7.35 ± 1.22	7.61 ± 1.29	6.92 ± 0.88	7.13 ± 1.19	7.29 ± 0.79
Serum parameters					
Creatinine (mmol/L)	25.33 ± 7.05	98.36 ± 11.17^a^	46.41 ± 8.28^ab^	29.53 ± 9.15^bc^	27.39 ± 5.81^bc^
Urea (μmol/L)	6.93 ± 1.55	24.96 ± 5.69^a^	12.74 ± 3.81^ab^	7.53 ± 2.58^bc^	7.34 ± 2.93^bc^

Values are expressed as the means ± standard error; ten animals in each group. (a) *p* <.05 versus group 1; (b) *p* <.05 versus group 2; (c) *p* <.05 versus group 3 and (d) *p* <.05 versus group 4.

The serum creatinine and blood urea nitrogen were significantly increased in group 2 (*p* < .05) compared to group 1. However, after 2 g/kg/day potassium citrate administration for 28 days in group 3, the two parameters were significantly decreased compared with that of group 2 (*p* < .05). Additionally, compared with those of group 2 and 3, the two indicators in group 4 and group 5 were significantly decreased after *Angelica sinensis* polysaccharide administration (*p* < .05).

### Serum and renal tissue oxidative stress levels

**Figure 2.**
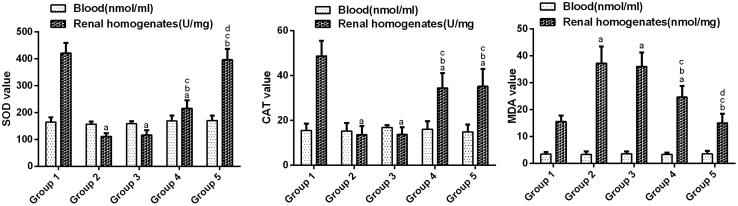
Oxidative studies. Values are expressed as the mean ± standard error (*n* = 10). (a) *p* <.05 versus group 1; (b) *p* < .05 versus group 2; (c) *p* < .05 versus group 3 and (d) *p* < .05 versus group 4.

Based on the serum oxidative stress values shown in Figure 2, the oral administration of ethylene glycol in rats at the dose 1% for 24 days did not show any sign of increased oxidative stress compared to group 1 and there was no increase for other group.

However, in the kidney, the SOD and CAT values of group 2 significantly decreased compared with group 1 (*p* < .05). The SOD and CAT values of group 3 was not significantly improved compared with those of group 2 (*p* > .05). The renal SOD levels in group 4 were significantly improved compared with that of group 2 (*p* < .05). The SOD levels were increased significantly in group 5 in kidney samples compared with those of group 4 (*p* < .05). The renal CAT levels in groups 4 and 5 were significantly improved compared with that of group 2 (*p* < .05). However, there was no obvious difference between the two groups.

In addition, the MDA values of group 2 were significantly the highest (*p* < .05). The MDA value of group 3 was not significantly lower than that of group 2 (*p* > .05), whereas it was still significantly higher than the values of group 1 (*p* < .05).However, after the treatment with different doses of *Angelica sinensis* polysaccharide, the MDA values in group 4 and group 5 decreased significantly in the kidney samples compared with those of group 2 and group 3 (*p* < .05) and the MDA values in group 5 decreased significantly in the kidney samples compared with those of group 4.

### Pathological changes

**Figure 3.**
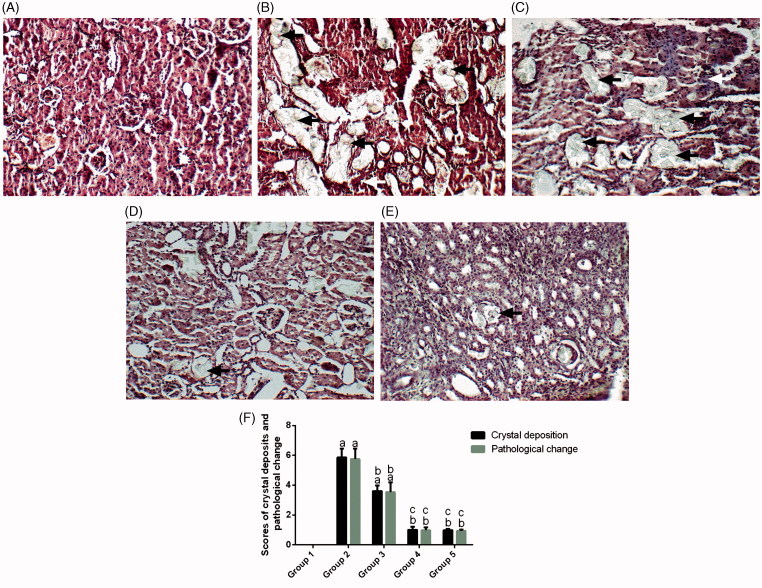
Histopathology (100×) of crystalline formation in the renal sections of rats with H&E staining for groups 1–5 (A–E) and scores for the crystal deposition and histopathological changes (F). Black arrows indicate renal crystals and white arrows indicate inflammatory cell invasion. Values are expressed as the mean ± standard error.(*n* = 10). a *p* <.05 versus group 1; b *p* <.05 versus group 2; c *p* < .05 versus group 3 and d *p* < .05 versus group 4.

Figure 3(B) indicates that the administration of 1% ethylene glycol for 28 days caused severe glomerular damage, tubular dilation, hyaline casts, membrane damage and interstitial inflammation (red arrow). In group 3, treatment with potassium citrate at 2 g/kg/day for 28 days clearly ameliorated the renal damage caused by ethylene glycol. The kidney histopathological scores indicated reduced renal damage as suggested by less interstitial inflammation and tubular dilatation in all the *Angelica sinensis* polysaccharide*-*treated rats (Figure 3(D,E)) compared with groups 2 and 3 (Figure 3(F)).

### Crystal deposition

As shown in Figure 3, the kidney sections of group 1 revealed the absence of CaOx deposits or other abnormalities in different segments of the nephrons. In group 2, all the kidney sections stained by H&E, including the cortex, medulla and papilla, displayed CaOx crystalline deposits (black arrow). However, in groups 3–5, compared with group 2, the number of CaOx deposits were decreased (*p* < .05). In addition, the number of CaOx deposits in groups 4 and 5 was significantly decreased compared with that of group 3, although significant differences were not observed between the two groups.

### Immunohistochemical staining

**Figure 4.**
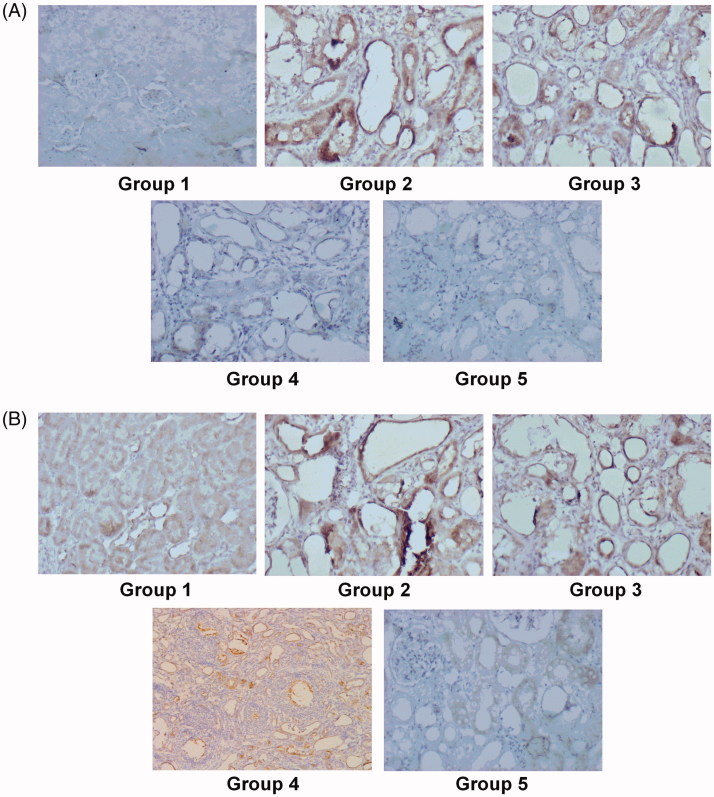
Immunohistochemical staining revealing the expression and location of KIM-1(A) and P-JNK (B) (100×).

To evaluate the mechanism by which *Angelica sinensis* polysaccharide attenuates renal crystal deposition, KIM-1/P-JNK signaling was examined by immunohistochemical staining. A high frequency and intensity of staining in KIM-1 was observed in the proximal tubule, distal tubule and collecting duct in the ethylene glycol-treated kidneys (Figure 4(A)); however, further faint KIM-1 staining was observed in the kidneys from the potassium citrate-treated urolithiasis rats (Figure 4(A)). In addition, the renal staining of KIM-1 in groups 4 and 5 was the weakest (Figure 4(A)). The P-JNK expression in all groups was similar to that of the KIM-1 protein (Figure 4B).To confirm these results, the changes were further examined by Western blot analysis.

### Western blot

**Figure 5.**
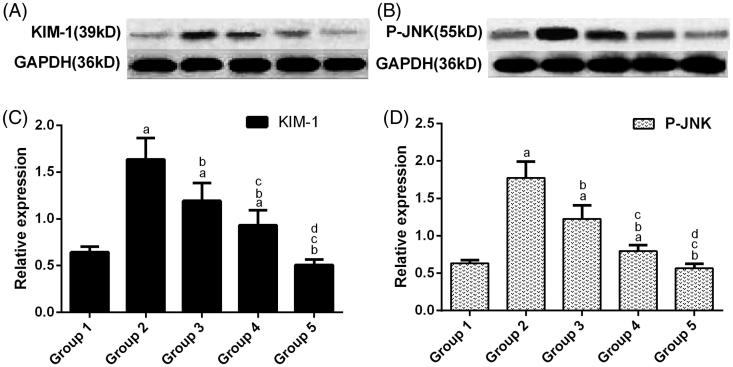
Representative immunoblots of the protein expression levels of KIM-1 and P-JNK. GAPDH from the samples was designated as the internal control. Values are expressed as the mean ± standard error (*n* = 3). (a) *p* < .05 versus group 1; (b) *p* < .05 versus group 2; (c) *p* < .05 versus group 3 and (d) *p* < .05 versus group 4.

Western blotting revealed that 1% ethylene glycol could significantly increase KIM-1 and P-JNK expression to varying high degrees compared with group 1(*p* < .05). When the ethylene glycol-treated rats were treated with 2 g/kg/day of potassium citrate for 28 days, the KIM-1 and P-JNK expression decreased significantly compared to group 2 (*p* < .05). However, in groups 4 and 5, the degrees of reduction in the expression of the two proteins were all significantly greater than those of group 3 (*p* < .05).Furthermore, when compared with that of group 4, group 5 showed the lower expression (Figure 5(B)).

## Discussion

The ability to hinder the crystal aggregation process *in vitro* is the primary criterion for a drug to be considered an anti-urolithic agent and bearing carboxylate side chains is a desirable prerequisite. In our study, *Angelica sinensis* polysaccharide inhibited calcium oxalate crystal growth, which is consistent with the results of a crystallization study, in which *Angelica sinensis* polysaccharide exhibited much greater inhibition than potassium citrate, which is a well-known calcium chelator [19]. The mechanism underlying the inhibition of the crystallization process was likely the reduction in calcium depletion after the addition of *Angelica sinensis* polysaccharide.

The results of the present study were consistent with our hypothesis that *Angelica sinensis* polysaccharide at doses of 80 and 320 mg/kg/day will inhibit CaOx crystal formation, as determined by the severity of renal crystal deposition. Moreover, the *Angelica sinensis* polysaccharide treatment groups presented significantly less CaOx crystal formation than the potassium citrate group. To the best of our knowledge, this is the first evidence supporting the inhibitory effect of *Angelica sinensis* polysaccharide on CaOx crystal formation in ethylene glycol-fed rats.

Many animal models have been established to study the mechanisms involved in the formation of CaOx urolithiasis and to ascertain the effect of various therapeutic agents on the development and progression of the stones [20]. Rats are the most frequently used animals in models of renal CaOx deposition, a process that mimics the etiology of urolithiasis formation in humans. Rat models of CaOx urolithiasis induced by ethylene glycol alone are often used to study the pathogenesis of renal crystal deposition [21]. Hepatic enzymes metabolize ethylene glycol to oxalic acid, which combines with calcium to form calcium oxalate crystals. This process can result in several biochemical abnormalities, including hypercalciuria, hypocitraturia, proteinuria, hyperoxaluria, hyperuricosuria, increased creatinine and renal failure in the lithic rats [22]. Additionally, several studies have shown that exposure to high oxalate and CaOx crystals can induce the production of reactive oxygen species (ROS) by the renal epithelial cells. ROS can mediate the urolithiasis-related expression of specific genes and protein synthesis and lead to renal epithelial injury, which is involved in crystal deposition [23,24], In the present study, we found that ethylene glycol could increase ROS levels, oxalate (the urinary stone-forming constituent) contents, P-JNK and KIM-1 expression, and these findings are consistent with previous results. However, hypercalciuria was not observed in group 2, as previously described. A possible explanation for this result is that the formation of CaOx consumes free calcium (Ca) and normalizes Ca levels in the urine.

Citrate is a potent inhibitor of stone formation—by complexing with calcium, citrate prevents crystallization by inhibiting the crystal growth of calcium phosphate and calcium oxalate, retarding the agglomeration of preformed calcium oxalate crystal and preventing the heterogeneous nucleation of calcium oxalate; monosodium citrate also restores the inhibitory properties of Tamm–Horsfall protein [25,26]. To inhibit stone formation, potassium citrate and other forms of alkaline citrate have been prescribed to stone-formers; when ingested orally, alkaline citrate has been shown in multiple studies to increase the urinary pH, increase urinary citrate, and significantly reduce the recurrence of calcium stones [27]. In our experiments, we found that urinary citrate levels were significantly increased in group 3 compared with group 1, which is consistent with previous study results. Moreover, we found that KCit inhibited ethylene glycol-induced urolithiasis-related P-JNK and KIM-1 expression, crystal deposition, and pathological changes and that it improved the renal functions in ethylene glycol-induced rats.

Abundant herbal drugs with antilithic effects that are used for treatment are available [28–30]. Some drugs have been studied using *in vitro* models such as nucleation, crystal aggregation and crystal growth [31]. In the present study, we assessed the preventive effects of an orally administered *Angelica sinensis* polysaccharide aqueous extract on CaOx urolithiasis in ethylene glycol-treated rats. After oral treatment with the *Angelica sinensis* polysaccharide aqueous extract, we observed an improvement in the urinary biochemistry and renal function; a decrease in ROS, P-JNK and KIM-1 expression; and reduced renal depositions compared with that of the ethylene glycol-induced rats.

A disturbance in the physiochemical milieu leads to ROS production and the development of oxidative stress. ROS start a signaling cascade culminating in the production of macromolecules to inhibit crystal nucleation, growth and aggregation. In the case of a transitory disorder, either no crystals will form or the crystals formed will remain small and well dispersed and be passed as crystalluria particles. In the face of a persistent disorder, for example, hyperoxaluria, hypercalciuria and hypocitraturia, the balance between oxidative and antioxidative forces is lost. ROS-induced damage to the cells leads to cell death and the formation of membrane bound vesicles, which support crystal nucleation [32,33].

High levels of oxalate and CaOx crystals lead to cellular injury, increased ROS levels and lipid peroxidation [34]. Lipid peroxidation has been suggested to be a predisposing factor for subsequent CaOx crystal deposition [35]. In this study, the antioxidant potential of an aqueous extract in the kidneys of rats was evaluated by lipid peroxidation inhibitory activity. A significant increase in the MDA contents and a significant decrease in the SOD and CAT levels, which is representative of lipid peroxidation, were observed in the kidneys of rats treated with ethylene glycol compared with those of the intact control group. Animals receiving aqueous extracts of *Angelica sinensis* polysaccharide exhibited lower lipid peroxidation levels than those of group 2. The exact mechanism(s) by which *Angelica sinensis* polysaccharide exerts its protective effects against ethylene glycol-induced urolithiasis is not yet understood, although it may be related to the flavonoids in the extract, which protect the kidney against the damaging effects of ROS, including peroxynitrite, peroxyl radicals, superoxide, singlet oxygen and hydroxyl radicals [36].

The JNK pathways play a critical role in response to cellular stress by promoting cell growth and survival [37]. In our present study, we investigated the JNK pathways in urolithiatic rats by directing antibodies against phosphorylated forms of JNK. Our results demonstrate that phosphorylated JNK was stimulated in urolithiatic rats. JNK could be activated by osmotic stress and during ischemia-reperfusion of the kidney [38]. Activation of the kinase pathway yields a plethora of changes in cell surface receptor synthesis, cytoskeletal structure, protein expression, and transcription, ultimately affecting cell survival and thereby leading to programed cell death and apoptosis [39]. Additionally, cellular death may cause the adherence of more crystals to the surfaces of renal tubular epithelial cells and increased crystal formation. Thus, any molecule that can modulate renal phosphorylated JNK expression can be expected to regulate the retention of calcium oxalate crystals in the tissue. In the present study, the phosphorylated JNK protein levels were reduced in the nephrolithiatic rats after the administration of *Angelica sinensis* polysaccharide at doses of 80 and 320 mg/kg/day.

In the normal kidney, (KIM-1 ‘a transmembrane glycoprotein’ typically cannot be detected. However, during the early stages of nephrotoxicity-induced or ischemic proximal tubule injury, KIM-1 may be upregulated and excreted in the urine [40,41]. In the majority of proteinuric, toxic and ischemia-associated renal diseases, KIM-1 is a marker. Due to its potential role in the modulation of tubular damage and repair, researchers pay great attention to it. KIM-1 may participate in restoring the renal tubular epithelium tissue, participate in maintaining its functional integrity and may also be involved in the proliferation and differentiation of proximal tubule endothelial cells [42,43]. In our study, KIM-1expression was also reduced relative to the nephrolithiatic rats when *Angelica sinensis* polysaccharide was administered at doses of 80 and 320 mg/kg/day.

Moreover, the rats in groups 4 and 5, which presented nearly normal histology’s, crystal deposit levels, protein expression and renal function, had lower crystal deposits, urolithiasis-related protein expression and renal function levels relative to the rats in group 3. Despite the administration of potassium citrate, the urinary oxalate and kidney oxidative stress levels of the rats in group 3 were still high. Thus, compared with potassium citrate, *Angelica sinensis* polysaccharide may be a better antilithiatic drug.

## Conclusion

The results of this investigation indicate that *Angelica sinensis* polysaccharide can effectively reduce crystal deposition in renal tubules in experimentally induced urolithiasis. *Angelica sinensis* polysaccharide may exert this effect through an ROS modulatory effect, which successively coordinates the expression of P-JNK and KIM-1 in the kidneys of rats. Moreover, this natural agent decreased urinary oxalate stone-forming constituents. The *Angelica sinensis* polysaccharide treatment was found to be more effective than the potassium citrate therapy used in the management of urolithiasis. *Angelica sinensis* polysaccharide offers promising potential as an antilithiatic agent in rats, and further research is warranted to elucidate the effect of Angelica sinensis polysaccharide in a patient diagnosed with a kidney stone.

## Abbreviations

CaOx: calcium oxalate
SOD: superoxide dismutase
ROS: reactive oxygen species
SD: Sprague-Dawley
MDA: malondialdehyde
CAT: catalase
KIM-1: kidney injury molecule-1
P-JNK: phospho-c-Jun NH2-terminal protein kinase
